# Association between miR-199a rs74723057 and MET rs1621 polymorphisms and the risk of hepatocellular carcinoma

**DOI:** 10.18632/oncotarget.13033

**Published:** 2016-11-02

**Authors:** Qianqian Wang, Xiangyuan Yu, Qiang Li, Linyuan Qin, Shengkui Tan, Xiaoyun Zeng, Xiaoqiang Qiu, Bo Tang, Junfei Jin, Weijia Liao, Moqin Qiu, Lijun Tan, Gaofeng He, Xiaomei Li, Songqing He, Hongping Yu

**Affiliations:** ^1^ Department of Epidemiology and Health Statistics, Guangxi Medical University, Nanning 530021, China; ^2^ Department of Epidemiology, School of Public Health, Guilin Medical University, Guilin 541004, China; ^3^ Laboratory of Hepatobiliary and Pancreatic Surgery, The Affiliated Hospital of Guilin Medical University, Guilin 541001, China; ^4^ Guangxi Key Laboratory of Molecular Medicine in Liver Injury and Repair, Guilin Medical University, Guilin 541001, China

**Keywords:** hepatocellular carcinoma, miR-199a, MET, single nucleotide polymorphism, risk

## Abstract

MicroRNAs (miRNAs) can regulate gene expression at post-transcriptional levels, thereby influence cancer risk. The aim of the current study is to investigate association between miR-199a rs74723057 and MET rs1621 and HCC risk in 1032 HCC patients and 1060 cancer-free controls. These two SNPs were genotyped by using the Agena MassARRAY genotyping system. Odds ratio (OR) and 95% confidence interval (95%CI) were calculated to assess the strength of the associations. We found that compared with the wild-type AA genotype of MET rs1621, the variant GG genotype was associated with a decreased risk for HCC (OR = 0.24, 95% CI = 0.06–0.96, *P* = 0.043). No association between miR-199a rs74723057 and HCC risk was observed. In addition, an interaction effect on HCC risk between the selected two SNPs was found. Among those who carried the CG/GG genotypes of miR-199a rs74723057, those who carried the GG genotype of MET rs1621 had a reduced risk of HCC, when compared with those who carried the AG/AA genotypes of MET rs1621 (OR = 0.15, 95% CI = 0.03~0.73, *P* for interaction = 0.018). Our results suggest that MET rs1621 polymorphism, alone and combined with miR-199a rs74723057, may influence susceptibility to HCC. Further large-scale association studies and functional studies are needed to validate our findings.

## INTRODUCTION

Liver cancer is one of the most common malignant tumors in the world. Hepatocellular carcinoma (HCC) accounts for 85% to 90% of the primary liver cancer. China is a country with a high incidence of liver cancer. It was estimated that approximately 50% of the new cases and death cases in world occurred in China [1]. The etiology of HCC is multifactorial, and chronic hepatitis B virus (HBV) infection is a major cause of HCC. However, the fact that only a small proportion of patients with chronic HBV infection eventually develop HCC suggests that genetic predisposition may play an important role in the development of HCC [2–4].

MicroRNAs (miRNAs) are small (20–25 nucleotides) non-coding RNAs, which degrade or inhibit the translation of their target messenger RNAs (mRNAs) by pairing with the 3′untranslated region (3′UTR) of mRNAs, thus regulate gene expression at post-transcriptional levels [5–7]. Compelling evidence indicates that miRNAs play an essential role in a variety of biological pathways including proliferation, differentiation, apoptosis, as well as various cancers [8, 9]. MiR-199a is an important regulatory factor, which has the similar function as tumor suppressors. Dysregulated expression of the miR-199a-3p and miR-199a-5p has been reported in many different types of human cancers including HCC [10–14]. It has been reported that miR-199a was linked to many target genes, such as MET, mTOR, and DDR1 [13, 15, 16]. MET proto-oncogene is involved in the process of cell proliferation, movement, differentiation and angiogenesis by combining with hepatocyte growth factor (HGF) [17–19]. Many studies have reported that MET is overexpressed in a variety of human cancers including liver, lung, breast, and colon cancer [19–24]. Wang [24] et al. reported that transgenic mice that overexpressed MET in hepatocytes spontaneously developed HCC, and when the transgene was inactivated, tumors regressed even at advanced stages of tumor progression, indicating that MET is involved in the development and process of HCC.

Sing nucleotide polymorphisms (SNPs) in miRNAs and their target genes (miRNA-binding SNPs) might affect the expression of miRNAs or miRNA-binding interactions by influencing the miRNA-mRNA binding efficacy, thereby playing a role in the development and progression of cancers [25–27]. Previous studies have shown that miR-199a and MET are implicated in human cancers including HCC [10–14, 19–24]. However, as far as we know, no research has been reported to evaluate the effect of variants in MET and miR-199a on the risk of HCC. In this hospital-based case-control study, we performed the genotyping of miR-199a rs74723057 and MET rs1621 and assessed their associations with HCC risk in the population of South China.

## RESULTS

### Characteristics of cases and controls

The distributions of demographic characteristics of the study subjects are presented in Table 1. There were no significant differences in the distribution of age and gender between the cases and the controls (*P* > 0.05). However, the cases were more likely to be smokers, drinkers and HBV infection individuals (*P* < 0.001).

**Table 1:** Distributions of selected variables in HCC cases and cancer-free controls

Variables	Cases *n* (%)	Controls *n* (%)	*P*^a^
All patients	1032	1060	
Age (year)			0.105
<= 48	525 (50.9)	577 (52.4)	
> 48	507 (49.1)	483 (45.6)	
Sex			0.642
males	901 (87.3)	933 (88.0)	
females	131 (12.7)	127 (12.0)	
Smoking status			< 0.0001
never	653 (63.3)	892 (84.2)	
ever	379 (36.7)	168 (15.8)	
Alcohol use			< 0.0001
never	688 (66.7)	919 (90.8)	
ever	344 (33.3)	141 (13.3)	
HBV infection			< 0.0001
(−)	170 (16.5)	962 (90.8)	
(+)	862 (83.5)	98 (9.2)	

Note: HBV, hepatitis B virus; HCC, hepatocellular carcinoma;

a Two-sided chi-square test for distributions between cases and controls.

### Associations of miR-199a rs74723057 and MET rs1621with HCC risk

Genotype distributions of the two selected SNPs in cases and controls are summarized in Table 2. The genotype distributions of the two SNPs among the controls were in HWE (*P* = 0.225 for miR-199a rs74723057, *P* = 0.819 for MET rs1621). Compared with individuals carrying the wild-type genotype AA of MET rs1621, those individuals carrying the variant genotype GG had a decreased risk of HCC (OR = 0.24, 95% CI = 0.06–0.96, *P* = 0.043).

**Table 2:** Logistic regression analysis of association between miR-199a rs74723057 and MET rs1621 genotypes on HCC risk

Variants	Cases *n* (%)	Controls *n* (%)	*P*^a^	Adjusted OR (95% CI)^b^	*P*^b^
miR-199ars74723057					
GG	511 (49.5)	534 (50.38)	0.743	1	
CG	436 (42.2)	448 (42.26)		1.02 (0.77–1.35)	0.887
CC	85 (8.2)	78 (7.4)		0.97 (0.56–1.62)	0.901
METrs1621					
AA	802 (77.7)	853 (80.5)	0.154	1	
AG	222 (21.5)	195 (18.4)		1.33 (0.96–1.85)	0.087
GG	8 (0.8)	12 (1.1)		0.24 (0.06–0.96)	0.043

Note: HCC, hepatocellular carcinoma; OR, odds ratio; CI, confidence interval.

a Chi-square test of genotype distribution among cases and controls.

b Adjusted for age, sex, smoking status, alcohol use and HBV infection.

We then assessed whether there was a combination effect between the miR-199a rs74723057 and MET rs1621 polymorphisms on the risk of HCC (Table 3). Among those individuals who carried the variant genotype CC of miR-199a rs74723057, we did not find a change in HCC risk for those individuals who carried the variant genotype GG of MET rs1621 (OR = 0.76, 95% CI = 0.04–14.2). However, among those who carried the CG/GG genotypes of miR-199a rs74723057, those who carried the GG genotype of MET rs1621 had a decreased risk of HCC (OR = 0.15, 95% CI = 0.03~0.73, *P* for interaction = 0.018), when compared with those who carried the wild-type AG/AA genotypes of MET rs1621.

**Table 3:** The genotype combinations of the SNP-SNP interaction in miR-199a rs74723057 and MET rs1621 with HCC risk

Genotypes		Cases *n*	Controls *n*	Adjusted OR (95% CI)^a^	*P*^b^
miR-199a rs74723057	MET rs1621				
GG+CG	AA + AG	941	972	reference	
	GG	6	10	0.15 (0.03~0.73)	0.018
CC	AA + AG	83	76	reference	
	GG	2	2	0.76 (0.04~14.02)	0.85

Note: HCC, hepatocellular carcinoma; OR, odds ratio; CI, confidence interval.

a Adjusted for age, sex, smoking status, alcohol use and HBV infection

b *P*-value for interaction

## DISCUSSION

In the current case-control study with 1032 HCC patients and 1060 cancer-free controls, we investigated whether miR-199a rs74723057 and MET rs1621 polymorphisms are associated with the risk of HCC in the population of South China. Eventually, MET rs1621 A > G was associated with the risk of HCC was found. Compared with individuals carrying the wild-type genotype AA, those individuals carrying the variant genotype GG had a decreased risk of HCC. Furthermore, we found that the two selected SNPs had a combination effect on the risk of HCC. Among those who carried the CG/GG genotypes of miR-199a rs74723057, those who carried the variant GG genotype of MET rs1621 had a decreased risk of HCC, when compared with those who carried the AG/AA genotypes of MET rs1621. Our results suggested that MET rs1621 polymorphism, alone and combined with miR-199a rs74723057 polymorphism, may influence susceptibility to HCC in the population of South China. To the best of our knowledge, this is the first study to investigate association between MET SNPs and HCC risk.

MET, a transmembrane receptor tyrosine kinase, is located at a region on chromosome 7q31 [28]. It is activated by its ligand HGF, thus playing an important role as a dominant oncogene in tumor development and progression [29]. The expression of MET has been shown to be overexpressed and correlated with poor prognosis in a number of major human cancers including HCC. It has been found that compared with the surrounding normal liver tissue, MET was overexpressed in HCC, suggesting that MET may contribute to the development of HCC [19, 20]. Ueki et al. found that HCC patients with high MET expression had a shorter 5-year survival than those with low MET expression [20]. Recently variants in MET have been reported to be associated with the prognosis of various human cancers, such as lung cancer and gastric cancer [30, 31]. Cao [30] et al. found that compared with small cell lung cancer (SCLC) patients who carried the CT/TT genotypes of MET rs41736, those SCLC patients who carried the wild-genotype CC tended to have a shorter progression-free survival and overall survival. This finding suggests that MET rs41736 could be considered as a prognostic marker for SCLC. Sunakawa [31] et al. found that compared with gastric cancer (GC) patients carrying the AA genotype of MET rs40239, those GC patients carrying the GG/AG genotypes of MET rs40239 had a longer disease-free survival and overall survival. In the current study, we found that the variant homozygous genotype GG of MET rs1621(A > G) was associated with the decreased risk of HCC.

MiRNAs can inhibit the majority of mRNA transcripts by post-transcriptional regulation. Now evidence has shown that miRNAs may contribute to various physiological processes and are involved in the development of HCC [10, 32]. It has been found that miR-199a expression was downregulated in HCC tissues when compared with adjacent non tumor tissues, and lower expression of miR-199a was correlated with low survival and poor prognosis of HCC [14, 33]. It was reported that MiR-199a-3p may inhibit HepG2 cell growth and invasion, and sensitize HepG2 cells to doxorubicin treatment and to hypoxia-induced apoptosis [15]. In addition, Shen [13] et al. found that miR-199a-5p could inhibit HCC cells invasion by binding to discoidin domain receptor 1(DDR1). A number of studies have demonstrated that SNPs in miRNAs may affect miRNA function and be involved in cancer development [34–36]. For example, Zhang [35] et al. investigated the association between miRNA196a-2 rs11614913C > T and HCC risk in a large Chinese case-control study, and they found that compared with individuals carrying the CC genotype of rs11614913, those individuals carrying the CT/TT genotypes showed decreased risk for HCC. Ma [36] et al. found that compared with subjects carrying the TT genotype of miR-499 rs3746444, those subjects carrying the CT/CC genotypes had a higher risk of HCC. In the current study, we investigated the association between miR-199a rs74723057 and the risk of HCC, but we failed to find that miR-199a rs74723057 alone was significantly associated with HCC risk. However, when we performed acombined analysis between the miR-199a rs74723057 and MET rs1621 polymorphisms on the risk of HCC, we found the combination effect of the two selected SNPs on the risk of HCC. Among those who carried CG/GG genotypes of miR-199a rs74723057, those who carried the variant genotype GG of MET rs1621 had a decreased risk of HCC, when compared with those who carried the AG/AA genotypes of MET rs1621. Our findings suggested that MET rs1621 polymorphism, alone and combined with miR-199a rs74723057, may influence susceptibility to HCC. MET is predicted to a direct target of miR-199a by a computational tool of SNPinfo (http://snpinfo.niehs.nih.gov/snpinfo). It was reported that miR-199a-3p can inhibit the cell cycle modulation of HCC cells by repressing the translation of mTOR and MET [15]. Furthermore, miR-199a can suppress both MET and its downstream effector ERK2, thus inhibit not only cell proliferation but also motility and invasive capabilities of tumor cells [16]. miRNA SNPs and miRNA-binding SNPs are likely to affect the expression of the target genes, and may contribute to individual susceptibility to human cancers [34, 37, 38].

The limitations of the study should be pointed out. First, our study was a hospital-based case-control study with potential selection bias. Second, our study was not powered enough to detect the interaction effects. Third, our findings were not mechanically validated.

In conclusion, our findings suggest that MET rs1621 polymorphism, alone and combined with miR-199a rs74723057, may influence susceptibility to HCC. Further large-scale association studies and functional studies are needed to validate our findings.

## MATERIALS AND METHODS

### Study population

All newly and histologically confirmed HCC patients were consecutively recruited from The First Affiliated Hospital of Guangxi Medical University, Affiliated Tumor Hospital of Guangxi Medical University and Affiliated Hospital of Guilin Medical University during the period from January 2007 to April 2011. All cases had not been treated with radiation, chemotherapy, or surgical therapy before enrollment in the study. Cancer-free controls were recruited from the same hospitals during the same period, which were genetically unrelated to the cases and were frequency matched to the cases on age (± 5 years) and sex. All subjects were interviewed to collect demographic data and history of environmental exposure after informed consent. Individuals who had smoked more than 6 months continuously or cumulatively in their lifetimes were defined as “ever smokers” and the rest as “never smokers”. Those subjects who had drunk alcoholic beverages at least once a week for more than 6 months were “defined as ever drinkers” and the rest as “never drinkers”. After interview, 5 ml of peripheral blood sample were collected from each subject. Finally, 1032 HCC cases and 1060 cancer-free controls were included in this study. This study was approved by institutional review committee board of Guilin Medical University.

### SNP selection and genotyping

Candidate SNPs, MiR-199a rs74723057 and MET rs1621, were selected based on the NCBI dbSNP database (http://www.ncbi.nlm.nih.gov/projects/SNP) and NIEHS SNPinfo (http://snpinfo.niehs.nih.gov/snpinfo). SNP rs74723057 is located at the promoter region of miR-199a, which may influence miR-199a expression by altering transcriptional activity. SNP rs1621 is located in the binding sites of miR-199a in the 3′ untranslated region (3′UTR) of MET gene, which may affect the miR-199a binding site activity. Genomic DNA was extracted from peripheral white blood cells by phenol–chloroform extraction and stored at −80°C. Genotyping of miR-199a rs74723057 and MET rs1621 polymorphisms was performed by using the Agena MassARRAY genotyping system (Agena; San Diego, CA) according to the manufacturer's instructions. The primers used for PCR were listed in Table 4. Each PCR reaction mixture contained 10 ng of genomic DNA, 0.5 uL 10 × PCR Buffer (with 15 mM MgCl_2_), 0.4 ul 25 mM MgCl_2_, 0.1 ul 25 mM dNTPs, 1 ul 0.5 uM primer Mix, and 0.2 ul 5 U/ul Hot Star Taq polymerase. Reaction was performed at 94°C for 15 min, followed by 45 cycles at 94°C for 20 s, 56°C for 30 s, and 72°C for 1 min, with a final incubation at 72°C for 3 min. Unincorporated dNTPs were deactivated by using 0.5 U of shrimp alkaline phosphatase (SAP) followed by primer extension using 0.2 ul iPLEX Buffer Plus, 0.2 ul iPLEX Termination mix, 0.94 ul iPLEX Extend Primer Mix, 0.041 ul iPLEX Enzyme (Agena; San Diego, CA). The extension reactions were performed at 94°C for 30 s and then 94°C for 5 s, followed by 40 cycles at 52°C for 5 s, 5 cycles at 80°C for 5 s, with a final incubation at 72°C for 3min. Purified extension reaction products were spotted onto a 384-well Spectro CHIPs and measured by using the platform MALDI-TOF mass spectrometry within the Agena MassARRAY system. Genotype calling was performed in real time with MassARRAY Typer software version 4.0 and analyzed by using the MassARRAY Typer software version 4.0.

**Table 4:** Primers used in the screening for SNPs by MassArray

Gene	SNP	Alleles	Primers(5′–3′)	Extension Primers(5′–3′)
miR-199a	rs74723057	G/C	ACGTTGGATGAAGCCCACTGC TTCATCCTG	ACGGTCTCCCCTTGCAAAGTCCT GACA
			ACGTTGGATGACAGTGACTGT GGAGAAGGC	
MET	rs1621	A/G	ACGTTGGATGGTAACCTACACC ACATGCAC	CAACCACATGCACTATACAGTAG
			ACGTTGGATGACCCTGAGCAG AACTTTGTG	

### Statistical analysis

Distributions of genotype frequencies in controls were tested by a chi-square goodness-of-fit test for Hardy–Weinberg equilibrium. A chi-square test was performed to make a comparison of the categorical variables, such as age, sex, smoking status, alcohol use, and HBV infection. The associations of each SNP with the risk of HCC were estimated by calculating the odds ratios (ORs) and their 95% confidence intervals (CIs) from multivariate logistic regression models. All analyses were two sided with a statistical significance set at a *P* value of < 0.05. All statistical analyses were performed using SPSS software, version 18.0 (SPSS Institute, Chicago, IL).
